# miR-449a promotes liver cancer cell apoptosis by downregulation of Calpain 6 and POU2F1

**DOI:** 10.18632/oncotarget.4821

**Published:** 2015-08-12

**Authors:** Yonglei Liu, Yutong Wang, Xiangjun Sun, Chuanzhong Mei, Liying Wang, Zengxia Li, Xiliang Zha

**Affiliations:** ^1^ Department of Biochemistry and Molecular Biology, Shanghai Medical College, Fudan University, Shanghai, China; ^2^ Department of Experimental Therapeutics, the University of Texas MD Anderson Cancer Center, Houston, TX, USA; ^3^ Research Center of Linyi People Hospital, Linyi, Shandong, China; ^4^ Department of Hepatobiliary Surgery, Linyi People Hospital, Linyi, Shandong, China

**Keywords:** liver cancer, proliferation, apoptosis, CAPN6, miR-449a

## Abstract

Our previous study shows that Calpain 6 (CAPN6) expression is regulated by PI3K-Akt in liver cancer through POU2F1 and CAPN6 which promote cell proliferation and inhibit apoptosis of liver cancer cells. microRNAs (miRNAs) plays important roles in regulation of gene expression. However, whether miRNAs regulates CAPN6 expression and its cellular function is still unknown. This study aims to investigate how miRNAs regulate liver cancer apoptosis through POU2F1-CAPN6. It was verified that POU2F1 could promote cell proliferation and inhibit apoptosis through CAPN6. Using methods of bioinformatics, miR-449a was predicted as a potential regulator of both CAPN6 and POU2F1. It was verified that CAPN6 and POU2F1 were the target genes of miR-449a by luciferase assay. CAPN6 and POU2F1 protein and mRNA levels decreased in liver cancer cells with miR-449a overexpression using western blot and real time RT-PCR assays. miR-449a expression was lower in liver cancer tissues compared with their normal ones, so did the cells. Overexpression of miR-449a inhibited cell proliferation, induced G1 phase arrest and cell apoptosis in liver cancer. Further research demonstrated that miR-449a inhibited cancer cell proliferation and induced apoptosis via suppressing both POU2F1 and CAPN6. The study indicated that miR-449a functions as a tumor inhibitor in liver cancer by decreasing POU2F1 and CAPN6 expression in liver cancer.

## INTRODUCTION

Liver cancer is one of the most common tumor, which is the third cause of cancer-related death worldwide [1]. The treatment of liver cancer is complex and complicated. Although hepatic resection and transplantation are relative effective therapeutic methods in liver cancer, tumor recurrence rate is still very high [2, 3]. Currently, there are no well-established effective adjuvant therapeutics for liver cancer [2]. There needs further research to elucidate the molecular mechanism of liver cancer for finding new therapy targets.

The calpains are a family of cysteine proteases, which involve in various biological processes such as cell proliferation, apoptosis, differentiation and others [4]. The family is classified into two groups including classical calpains and non-classcial calpains [4]. Calpain 6 (CAPN6) belongs to the latter and is widely expressed in tumor tissues [4–6]. Previous reports show that the cellular function of CAPN6 includes the regulation of cell microtubule dynamics and cytoskeletal organization in embryonic tissues [7–9]. It may involve in tumorigenesis such as inhibiting cell apoptosis and promoting angiogenesis [10]. However, its regulatory mechanism by microRNAs (miRNAs) is not known.

More and more miRNAs are discovered in human cells. miRNAs are noncoding small RNA containing about 22–29 nucleotides and inhibits gene expression on mRNA or protein levels by specifically binding and cleaving mRNAs [11, 12]. miRNAs may function as suppressors or onco-miRNAs in various types of cancer and half of them locate in cancer-associated genomic regions [13, 14]. There are a number of miRNAs have been reported to be dysregulated in human liver cancer tissues or cells, including miR-1246, miR-509–5p, miR-181a-5p, miR-122, miR-137 and etc [15–19]. These miRNAs play important roles in cell biological behavior by inhibiting the expression of their target genes.

Our previous report shows that CAPN6 is regulated by PI3K-Akt pathway and inhibits cancer cell apoptosis [20]. Here, we continue to investigate the regulation of CAPN6 and POU2F1 in liver cancer by miRNAs. CAPN6 and POU2F1 were predicted as the target genes of miR-449a by bioinformatics. The expression of miR-449a in human liver cancer cells and tissues was examined. Cellular function of miR-449a including cell growth, cell cycle, and apoptosis was analyzed in liver cancer cell lines and the underlying mechanism of miR-449a functions in liver cancer was also investigated.

## RESULTS

### Inhibition of POU2F1 promotes liver cancer cell apoptosis via CAPN6

**Figure 1:**
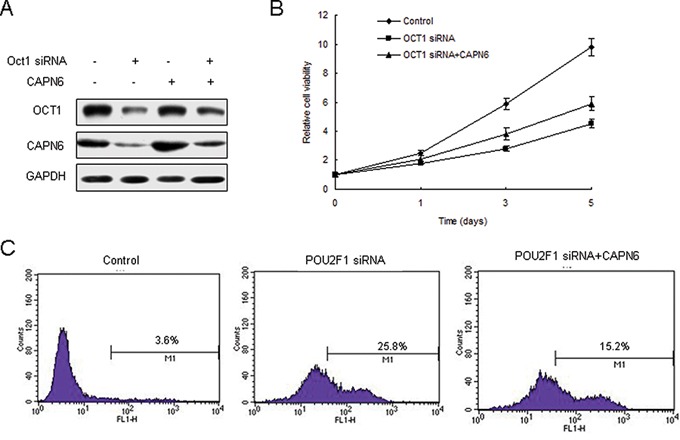
POU2F1 regulates liver cancer cells apoptosis via CAPN6 **A.** Inhibition of POU2F1 down-regulated CAPN6 expression in 7404 cells. 7404 were transfected POU2F1 siRNA or their control, and total protein was isolated for western blotting. **B.** Inhibition of POU2F1 could reduce cell viability of 7404 cells. **C.** Inhibition of POU2F1 promoted liver cancer cell apoptosis via CAPN6. 7404 cells were transfected POU2F1 siRNA or their control or combined with LV-CAPN6. The cells were strained with AnnexinV and analysed by flow cytometry.

We have demonstrate previously that POU2F1 regulates CAPN6 expression in liver cancer cells [20]. But the role of POU2F1 in cell apoptosis and proliferation by CAPN6 is not known. Firstly, 7404 cells were transfected POU2F1 siRNA and POU2F1 decreased, so did CAPN6 (Figure 1A). It was found that inhibition of POU2F1 suppressed cell proliferation of 7404 cells (Figure 1B). Analysis of flow cytometry showed that knocking down of POU2F1 induced apoptosis rate of liver cancer cell with CAPN6 overexpression to decrease (Figure 1C).

### miR-449a suppresses CAPN6 and POU2F1 expression in liver cancer cells

**Figure 2:**
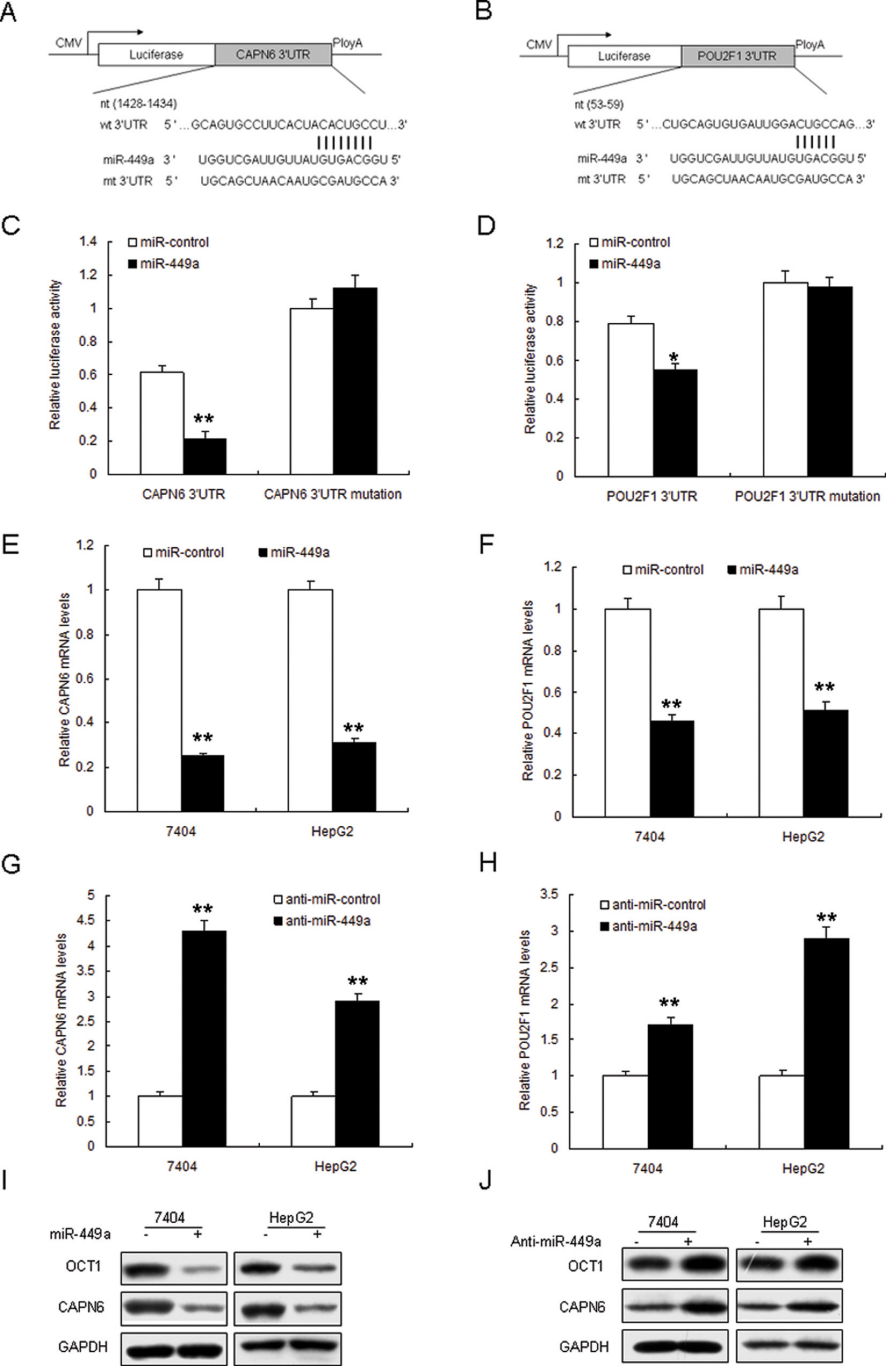
Restoration of miR-449a down-regulates POU2F1 and CAPN6 expression **A** and **B.** The 3′-UTR of the CAPN6 and POU2F1 genes contains binding sites for miR-449a according to bioinformatic analysis. **C** and **D.** miR-449a suppressed the expression of a luciferase reporter gene harbouring the 3′-UTR of CAPN6 or POU2F1. The pGL4 plasmid was modified by adding the human 3′-UTR or the 3′-UTR with mutations in regions complementary to miR-449a seed regions behind the firefly luciferase gene. HEK293T cells were transiently co-transfected with negative control (mock) or miR-449a together with the indicated luciferase constructs, and luciferase activity was analysed 48 h later. Data are presented as relative firefly luciferase activity normalized to Renilla luciferase activity from the same construct. **E** and **F.** miR-449a restoration down-regulated CAPN6 and POU2F1 in liver cancer cells. Cells were transfected with miR-449a or miR control for 48 hours, then collected for Real-time PCR. **G** and **H.** miR-449a restoration down-regulated CAPN6 and POU2F1 in liver cancer cells. Cells were transfected with miR-449a or miR control for 48 hours, then collected for Western blot analysis. **I** and **J.** miR-449a restoration down-regulated CAPN6 and POU2F1 in liver cancer cells. Cells were transfected with miR-449a or miR control for 48 hours, then collected for Western blotting. The data presented are shown as means ± s.d. collected from three independent experiments. *p < 0.05, **p < 0.01

The TargetScan database predicts that *CAPN6 and POU2F1* may be the target genes of miR-449a (Figure 2A and 2B). Data from luciferase assay showed that the luciferase activity of wide types of pGL3-CAPN6 and pGL3-POU2F1 in 7404 cells was much lower than the controls, and the luciferase activity of mutated pGL3-CAPN6 was rescued in 7404 cells (Figure 2C and 2D). Endogenous CAPN6 and POU2F1 expression in liver cancer cells with miR-449a overexpression were examined. The results showed that their mRNA decreased when 7404 and HepG2 cells were transfected with miR-449a (Figure 2E and 2F). CAPN6 and POU2F1 mRNA increased in the cells with anti-miR-449a (Figure 2G and 2H). POU2F1 and CAPN6 protein reduced in the cell with miR-449a and increased with anti-miR-449a (Figure 2I and 2J). Above data showed that CAPN6 and POU2F1 were direct target genes of miR-449a.

### Low miR-449a expression in human liver cancer

**Figure 3:**
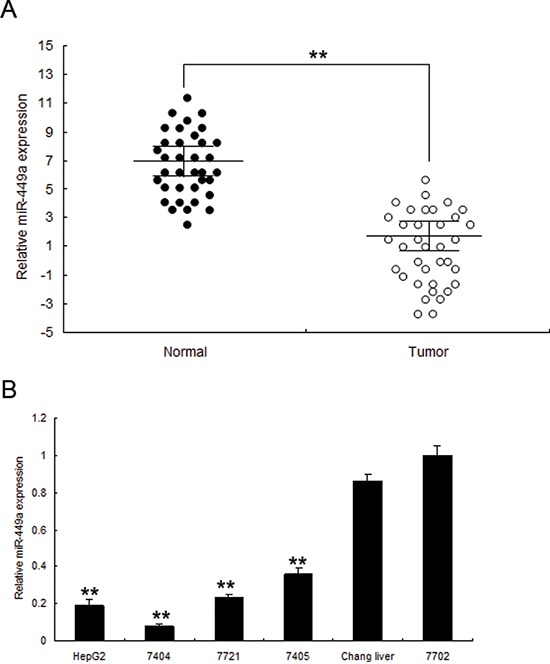
miR-449a is downregulated in human liver cancer tissues and cell lines **A.** miR-449a was lower in liver cancer tissues than the normal ones by immunohistochemistry. **B.** Real time PCR analysis of miR-449a in 7404, 7405, 7721, HepG2 cancer cells and normal cells changs liver, 7702. The data presented are shown as means ± s.d. collected from three independent experiments. **p < 0.01

In order to explore the cellular function of miR-449a in liver cancer, the expression of miR-449a was examined in human liver specimens by real time RT-PCR. miR-449a was lower in liver cancer tissues (*n* = 48) than the normal ones (*n* = 48) by real time RT-PCR (Figure S1 and 3A). Similarly, miR-449a was lower in four human liver cancer cell lines including HepG2, 7404, 7721 and 7405 compared with Changs liver and 7702 normal liver cell lines (Figure 3B). Relationship of miR-449a and clininic characteristics were shown in Table 1. These results suggested that miR-449a play a suppressing miRNA in liver cancer.

**Table 1:** Clinicopathologic correlations of miR-449a expression in liver cancer

	Case (n)	miR-449a level		*P* value
		Low (<Median)	High (>Median)	
**Sex**				
Male	42	27	15	0.271
Female	6	2	4	
**Age (years)**				
≤50	23	13	10	0.253
>50	19	11	8	
**Edmondson Grading**				
I+II	16	9	7	0.659
III+IV	32	18	14	
**Tumor Size (cm)**				
<5	14	8	6	0.562
≥5	28	13	15	
**Serum AFP (ng/ml)**				
≤20	11	5	6	0.412
>20	37	15	22	
**Serum HBsAg**				
Positive	28	13	15	0.628
Negative	20	12	8	
**Liver Cirrhosis**				
Positive	22	9	13	0.451
Negative	26	14	12	
**Distant Metastasis**				
Positive	6	4	2	0.407
Negative	42	20	22	
**Vascular Invasion**				
Positive	9	5	4	0.045
Negative	39	21	18	

### miR-449a suppresses liver cancer cell proliferation

**Figure 4:**
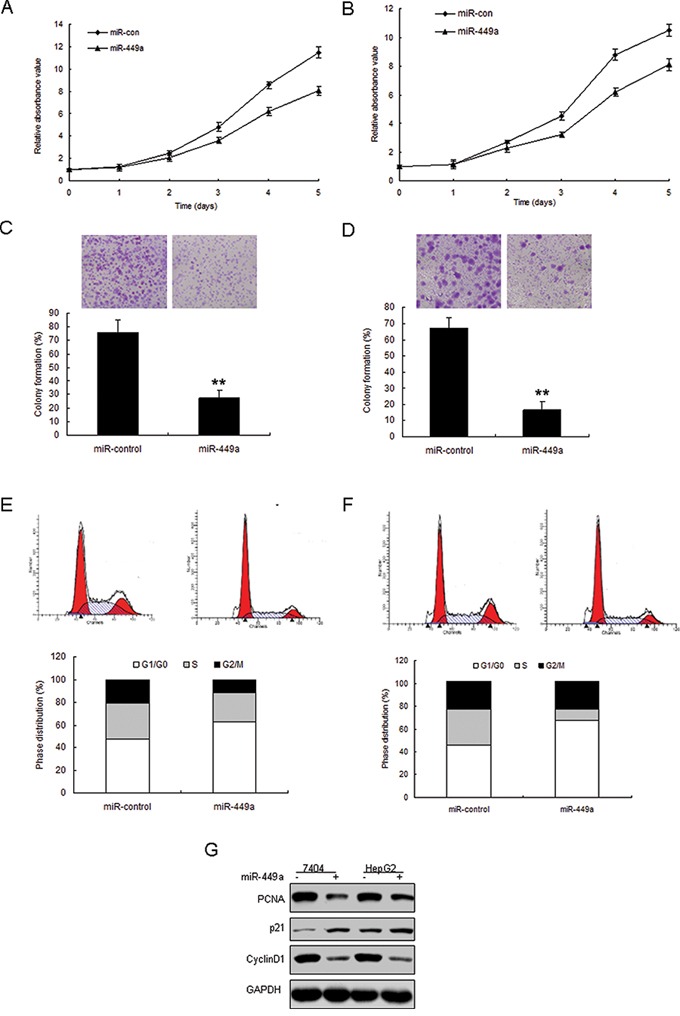
Enforced expression of miR-449a induces growth inhibition in liver cancer *in vitro* **A.** miR-449a expression in liver cancer cells was examined by real time RT-PCR. **B.** Effect of miR-449a on cell proliferation was measured by MTT assay after LV-miR-449a infection in HepG2 cells. **C.** Effect of miR-449a on cell proliferation was measured by colony formation assay after miRNA infection in 7404 cells. **D.** Effect of miR-449a on cell proliferation was measured by colony formation assay after miRNA infection in HepG2 cells. **E.** Cell-cycle distribution of 7404 cells infected with miRNAs. **F.** Cell-cycle distribution of HepG2 cells infected with miRNAs. **G.** 7404 and HepG2 cells were infected with miR-449a or the control, total protein was extracted for Western blotting. The data presented are shown as means ± s.d. collected from three independent experiments. **p < 0.01

In order to explore the possible role of miR-449a in cell growth, 7404 and HepG2 cells were transfected with miR-449a mimics (miR-449a) or its control (miR-control). The transfection effect was verified by real time RT-PCR and miR-449 expression was enhanced in the two cell lines (Figure S2). The results from MTT and colony formation assays indicated that miR-449a suppressed cell survival abilities and colony formation rates in 7404 cells (Figure 4A and 4B) and HepG2 cells (Figure 4C and 4D). To further observe miR-449a mediating growth inhibition, cells were transfected with miR-449a and analyzed the distribution of cell cycle. Compared with miR-control, 7404 and HepG2 cells with miR-449a overexpression showed an increasement of G1 phase and reduction of S phase (Figure 4E and 4F). These results suggested that miR-449a played an suppressing role in cell growth due to a G1-phase arrest. It was also found that cell proliferation associated protein PCNA decreased and cell cycle regulated proteins such as cyclinD1 was down-regulated and p21 was up-regulated in 7404 and HepG2 cells with miR-449 overexpression (Figure 4G).

### miR-449a inhibits proliferation and induced apoptosis of liver cancer cells by targeting POU2F1 and CAPN6

**Figure 5:**
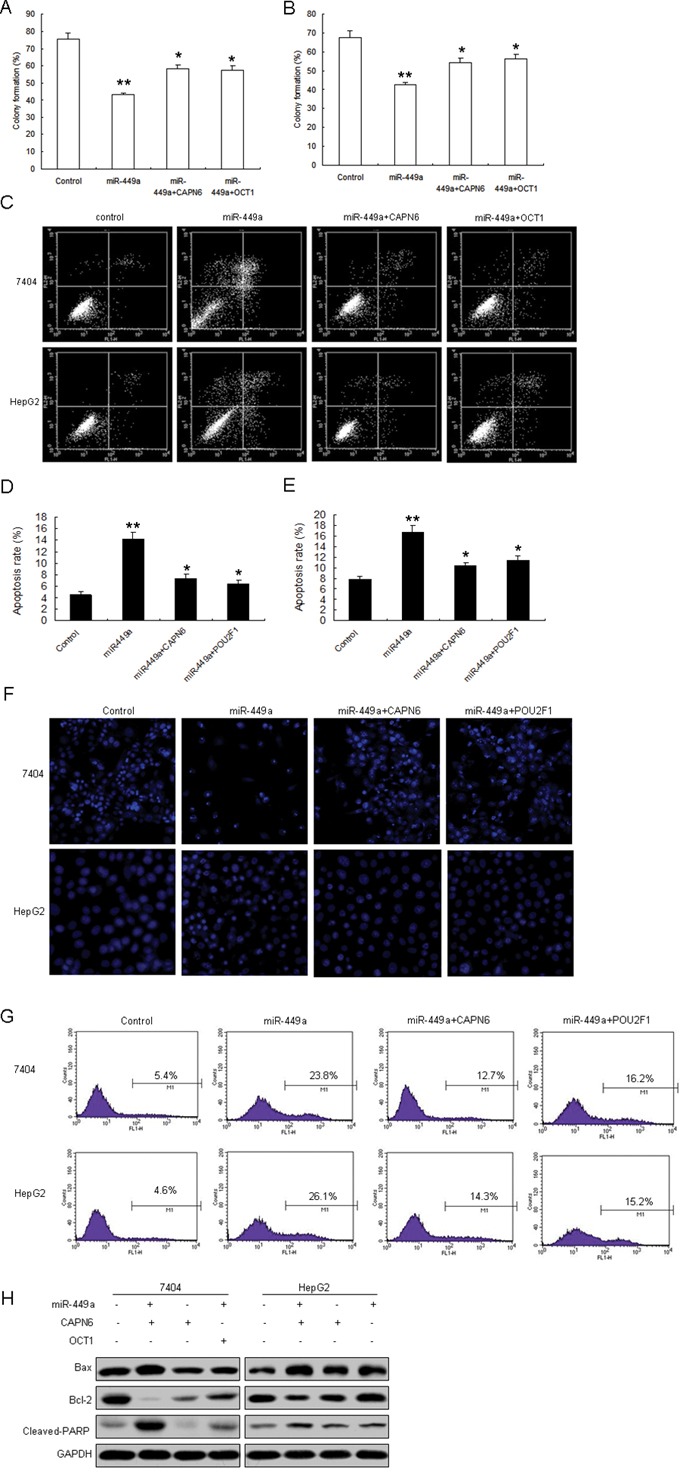
miR-449a inhibits cell proliferation and induces apoptosis of liver cancer cells by targeting POU2F1 and CAPN6 **A.** and **B.** 7404 and HepG2 cells were infected with miR-449a or CAPN6 or POU2F1, and cell proliferation was assayed by colony formation. **C.** miR-449a induced liver cancer cell apoptosis with CAPN6 overexpression. 7404 and HepG2 cells were infected with miR-449a or CAPN6 or POU2F1, and cell apoptosis rates were assayed by flow cytometry. **D.** Quantification of apoptosis rate of 7404 cells from C. **E.** Quantification of apoptosis rate of HepG2 cells from C. **F.** miR-449a induced liver cancer cell apoptosis with CAPN6 overexpression. 7404 and HepG2 cells were infected with miR-449a or CAPN6 or POU2F1, and cell apoptosis was assayed by Hoechst 33342 Straining. **G.** 7404 and HepG2 cells were infected with miR-449a or CAPN6 or POU2F1, and caspase 3 activity was assayed by flow cytometry. **H.** 7404 and HepG2 cells were infected with miR-449a or the control, total protein was extracted for Western blotting. The data presented are shown as means ± s.d. collected from three independent experiments. *p < 0.05, **p < 0.01

Next, given the fact that CAPN6 and POU2F1 promotes cell proliferation and induce apoptosis resistance in cancer cells, we want to know whether miR-449a suppresses cell proliferation and apoptosis in liver cancer cells by targeting POU2F1 and CAPN6. Data from colony formation assay showed that miR-449a in 7404 and HepG2 cells inhibited cell proliferation (Figure 5A and 5B). Cell apoptosis was increased in 7404 and HepG2 cells with miR-449a, when combined with CAPN6 or POU2F1 overexpression, apoptosis rate was decreased (Figure 5C, 5D and 5E). Hoechst staining showed that miR-449a could induce a significant apoptosis including nuclear fragmentation and chromosomal condensation in the liver cancer cells, however, in the cells with miR-449a and CAPN6 or POU2F1 overexpression, the apoptosis was attenuated (Figure 5F). There was a similar result from caspase 3 assay (Figure 5G). Western blotting analysis showed that miR-449a led to Bax increasing, and Bcl-2 decreasing and CAPN6 or POU2F1 overexpression in the cells could attenuated Bax expression and promoted Bcl-2 expression in 7404 and HepG2 cells (Figure 5H). These data clearly indicated that miR-449a promoted liver cancer cell apoptosis by down-regulation CAPN6 or POU2F1.

### miR-449a expression is negatively associated with CAPN6 and POU2F1 in liver cancer tissues

**Figure 6:**
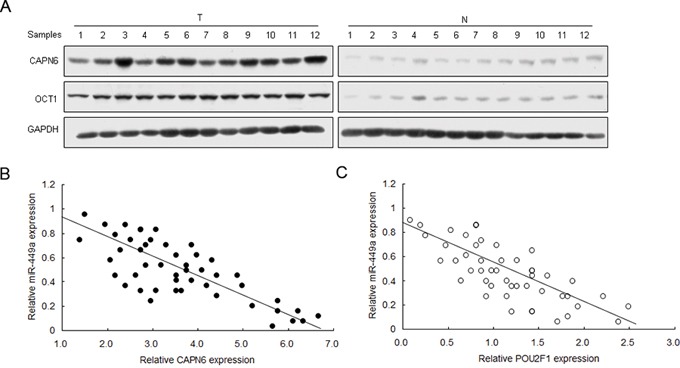
miR-449a expression is negatively associated with CAPN6 and POU2F1 in liver cancer tissues **A.** CAPN6 and POU2F1 protein were over-expressed in liver cancer tissues compared to their normal ones. **B.** and **C.** miR-449a was negatively associated with CAPN6 and POU2F1 protein in liver cancer. The data presented are shown as means ± s.d. collected from three independent experiments. *p < 0.05, **p < 0.01

To learn the relationship between miR-449a and CAPN6 or POU2F1 in liver cancer, CAPN6 and POU2F1 mRNA were analyzed by real time RT-PCR. CAPN6 and POU2F1 protein levels were overexpressed in liver cancer tissues compared with the normal ones (Figure 6A). CAPN6 and POU2F1 were positively associated with liver cancer (Supplemental Figure S3 and S4). Results of miR-449a expression was shown in Figure 1A. miR-449a was negatively associated with CAPN6 and POU2F1 in liver cancer (Figure 6B and 6C).

## DISCUSSION

CAPN6 is a potential oncogene in tumors and its regulation by miRNAs is still unknown. In our study, we investigated the molecular mechanism of CAPN6 regulation by miR-449a. Our data demonstrated that miR-449a inhibited liver cancer progression by targeting both CAPN6 and POU2F1. It was also found that miR-449a expression was greatly downregulated in cancer tissues of liver compared with that of normal ones and negatively associated with CAPN6 and POU2F1.

CAPN6 is overexpressed in many types of tumor and its function in tumor includes leading to apoptosis resistance, promoting cell proliferation and angiogenesis. Our reports indicated that PI3K-Akt regulates CAPN6 promoter activity, mRNA and protein via POU2F1 [20]. In this study, we carried out further research to investigate CAPN6 regulation by miRNAs. It was verified that CAPN6 was a new target gene of miR-449a and miR-449a could inhibit cell proliferation, induce G1 arrest and metastasis by down-regulation of CAPN6. Interestingly, it was also verified that POU2F1 was a target gene of miR-449a.

Our study showed that miR-449a expression was usually under-expressed in liver cancer tissues and cell lines, which is consistent with the previous reports that miR-449 expression was suppressed in liver cancer and other tumors and cancer cell lines [21–31]. Our data indicated that loss of miR-449a in liver cancer tissues was related to tumor progression such as tumor staging, which suggests that miR-449a may be a inhibitor of cancer metastasis. But our data in the cell lines were not significant (Figure S5). It is better to elucidate the relationship in many cell lines and more samples. We found that miR-449a induced G1 arrest of liver cancer cells, inhibited cell proliferation and promoted cell apoptosis, which were consistent with the reports from other tumors like gastric cancer. miR-449a is situated in the first intron of CDC20B on chromosome 5q11, and molecular mechanisms for down-regulation of miR-449a expression in liver cancer tissues and cell lines is still not known, one reason was that it was inhibited by histone deacetylases (HDAC)1–3 [31]. There needs further research to investigate it. Our results indicated that miR-449 regulates liver cancer growth acting as a suppressor, which could partially explain the relationship between the down-regulation of miR-449 expression and liver cancer' poor prognosis.

Reports from the researchers reveal that miR-449 expression is commonly lower in various tumors such as gastric cancers [21–22], endometrial cancer [23], lung cancer [24–25], colon cancer [26], prostate cancers [27–28] and others. There are many target genes of miR-449a are identified and cellular function of miR-449 involves inducing G1 arrest, apoptosis and senescence. Its target genes play important roles in cell cycle and apoptosis include Bcl-2 [21–22], CDC25A [22, 29], CDK6 [23, 29], E2F3 [25], c-Met [26], histone deacetylase 1 (HDAC1) [27–28] and E2F1 [30]. However, little is known about the role of miR-449a in liver cancer progression. A report showed that miR-449a decreased in hepatocellular carcinoma cells due to histone deacetylases (HDAC)1–3 up-regulation [31]. In our study, we identified that CAPN6 or POU2F1 are targets of miR-449a and there was an inverse correlation between miR-449a and CAPN6 or POU2F1 in liver cancer tissue specimens.

In a summary, we performed a series of experiments to reveal that miR-449a is a tumor inhibitory miRNA molecule in liver cancer, and low miR-449a expression was a potential marker for poor prognosis in patients with liver cancer. miR-449a has the influences on liver cancer cell behavior partially by down-regulation of CAPN6 and POU2F1. Our findings revealed that miR-449a is a potential value for liver cancer therapy and may be a good marker for prognosis prediction and diagnosis in liver cancer tumorigenesis. The study expanded the basic theory of pathogenesis of liver cancer and its possible therapeutic strategies.

## MATERIALS AND METHODS

### Cell culture

BEL-7404 (7404), BEL-7405 (7405), SMMC7721, HepG2 human liver cancer cell lines and Chang liver and HL-7702 (7702) normal liver cell lines were purchased from Shanghai Cancer Institute (China). HEK293T cells were stored in our lab. All the cell lines were maintained in the Dulbecco's Modified Eagle's medium (DMEM, Gibco, USA) supplemented with 10% fatal bovine serum (Gibco, USA) in a humidified incubator at 37°C and 5% CO_2_.

### Clinical specimens

Primary liver cancer samples and their adjacent ones from liver cancer patients were obtained from Zhongshan Hospital (Fudan University, Shanghai, China). Both tumor and their compared ones were histologically confirmed by H&E (hematoxylin and eosin) staining. Informed consent was obtained from each patient, and the research protocols were approved by the ethics committee of Fudan University.

### Plasmids and antibodies

CAPN6 primary antibody was obtained from Abcam (Cambridge, England). PCNA, p21, CyclinD1, Caspase3, PARP and POU2F1 primary antibodies were ordered from Cell Signaling (Danvers, MA, USA). GAPDH primary antibody and HRP labeled secondary antibodies were purchased from Kang-Chen Biotech (Kangcheng, Shanghai, China). CAPN6 plasmid was ordered from Origene (Rockville, MD, USA).

### Lentivirus mediating miR-449a

Vector carrying miR-449a was constructed using the BLOCK-iT pol II miR RNAi Expression Vector Kit with EmGFP (Invitrogen, Carlsbad, CA, USA). The primary miR-449a sequence with flanking regions was amplified by PCR. The sequence was cloned into pcDNA6.2-GW/EmGFP-miR. Lentivirus for miR-449a was produced using 293T cells by co-transfection with lipofectamine2000 (Invitrogen, Carlsbad, CA, USA) according to the protocol from the manufacturer. Cells were infected with the lentivirus and miR-449a expression was examined using real time RT-PCR.

### Transfection and dual luciferase assay

Cells were seeded on plates and transfected in Opti-MEM medium using lipofectamine2000 according to the manufacturer's protocol (Invitrogen, Carlsbad, CA, USA). The medium was changed after transfection for 5 h, and the cells incubated at 37°C for the indicated time. For reporter assays, cells were transfected with the pGL3-basic vector or the control plasmid with co-transfecting with siRNAs. 48 h later, luciferase activity was detected using Dual Luciferase Assay System (Promega, WI, USA) with a Sirius luminometer (Berthold Detection System).

### RNAi treatment

POU2F1 (NM_002697) siRNA was synthesized by Genepharma (Shanghai, China). The target sequences were the same to the previous report [20]. Approximately 2 × 10^5^ cells were seeded in 6-well plates and siRNA transfection was performed using lipofectamine 2000. The knockdown effect was checked using RT-PCR and western blotting.

### Real time RT-PCR

Total RNA was extracted from the cells with the indicated treatment using Trizol reagent (Invitrogen) according to the manufacturer's protocol. RNA was qualified and performed for real time RT-PCR analysis. The primers are as the following: POU2F1 sense: 5′-GAA GCC TTG AAC CTC AGC TTT-3′ and antisense: 5′-TCT CTA TGC TGG TGC GTT TCT-3′; CAPN6 sense 5′-ACT ATG GGT CCT CCT CTG-3′; antisense: 5′-AGC TGG TGG TTG CTA ATG-3′. GAPDH sense: 5′-ACC ACA GTC CAT GCC ATC AC-3′; anti-sense: 5′-CCA CCA CCC TGT TGC TGT AG-3′. The relative Mrna levels were calculated by comparing Ct values of the samples with those of the reference, all data normalized to the internal control GAPDH. MiRNA was reverse transcribed into Cdna using the TaqMan MicroRNA Reverse Transcription kit (Invitrogen). MiR-449a-specific primers (Forward: 59TGGCGGTGGCAGTGTATTGTTA39; Reverse: 59GTGCAGGGTCCGAGGT39) (Universal ProbeLibrary Probe #21, Roche, Germany), and KAPA PROBE FAST Qpcr Master Mix (KAPA BIOSYSTEMS, Boston, MA, USA).

### Western blotting

Briefly, total protein was extracted from the cells its concentration was determined by Lowry method. Total protein was separated by SDS-PAGE and then transferred to a PVDF membrane at 320 mA for 2 h at 4°C. The protein in the PVDF membrane was blocked with 5% fat-free milk in PBST, cultured in primary antibodies and then incubated in the secondary antibodies for about 1–2 h at room temperature. PVDF membranes were washed with PBST (3 × 10 min). The protein bands were visualized using ECL according to the the manufacturer's instructions (Promega).

### Cell cycle analysis

Liver cancer cells were transfected with POU2F1 siRNA, miR-449a or CAPN6 and collected for cell cyle analysis. The collected cells were stabilized with 75% ethanol for 24 h and washed in PBS and then dyed with PI. Cell cycle was analyzed with ModFit of flow cytometry.

### Colony formation assay

The liver cancer cells were plated in 6-well plates and transfected with the indicated miRNA, plasmid or siRNAs. 48 h later, the cells were collected and plated in 6-cm culture plates. The medium underwent the replacement at three-day intervals. And then the cells were fixed in 90% ethanol, stained with crystal violet and colonies consisting of at least 50 cells were counted ten days later.

### Determination of apoptosis

The liver cancer cells were plated in 6-well plates and transfected with the indicated miRNA, plasmid or siRNAs. The complete growth medium were changed to serum-free growth medium 24 h later and the cells were collected 24 h later. The cells were washed with cold PBS for two times, resuspended using 1 × binding buffer and adjusted to 10^6^ cells/ml. 10^5^ cells were taken out and 5 μl Annexin V-FITC and 5 μl PI (BD Biosciences, San Jose, CA, USA) were added into the cells, vortexed gently and incubated for 15 min at RT in the dark. Finally, 400 μl 1 × binding buffer was added to each tube. Cell apoptosis was analyzed by flow cytometry.

### Hoechst 33342 staining

Assay of Hoechst 33342 staining was used to visualize cell nuclear morphology and apoptosis. Cells were tranfected with miRNAs, siRNA or plasmids, treated with either Green 1 or NSC 51046, and then incubated with Hoechst 33342 dye (10 μM) (Molecular Probes, Eugene, OR, USA), culturing at 37°C for 10 minutes. The images of cell nuclear were got from using inverted fluorescence microscope (Leica DM IRB, Wetzlar, Germany) with 400X magnification.

### Caspase 3 activity

Cells were seeded in the 6-well plates and infected with lentivirus mediated miR-449a or POU2F1 or CANP6 for 48 h, and washed in cold 1X PBS for two times. Resuspend cells in BD Cytofix/Cytoperm™ solution were adjusted to 1 × 10^6^ cells in 500 ul buffer and incubated on ice for 20 min. Cells were pelleted, aspirated, discarded BD Cytofix/Cytoperm™ solution and washed in 1X BD Perm/Wash™ buffer at a volume of 1 × 10^6^ cells in 500 ul buffer at room temperature for two times. The cells were resuspended in the above buffer and incubated with the antibody for 30 min at room temperature. The cells were washed, resuspended in 0.5 ml 1XBD Perm/Wash™ buffer and then analyzed by flow cytometry.

### Statistical analysis

Data were analyzed by SPSS 13.0 software and presented as mean ± SE of at least three independent experiments. Two-tailed Student's *t* test was used for comparisons of two independent groups. *p* < 0.05 was considered statistically significant.

## SUPPLEMENTARY FIGURES


