# Supplementary material for: miR-449a promotes liver cancer cell apoptosis by downregulation of Calpain 6 and POU2F1
Source: Oncotarget. 2015 Aug 12;7(12):13491–501. doi: 10.18632/oncotarget.4821 (PMC4924656; doi:10.18632/oncotarget.4821)
Supplement: Supplementary file 1 [file oncotarget-07-13491-s001.pdf]

SUPPLEMENTARY FIGURES

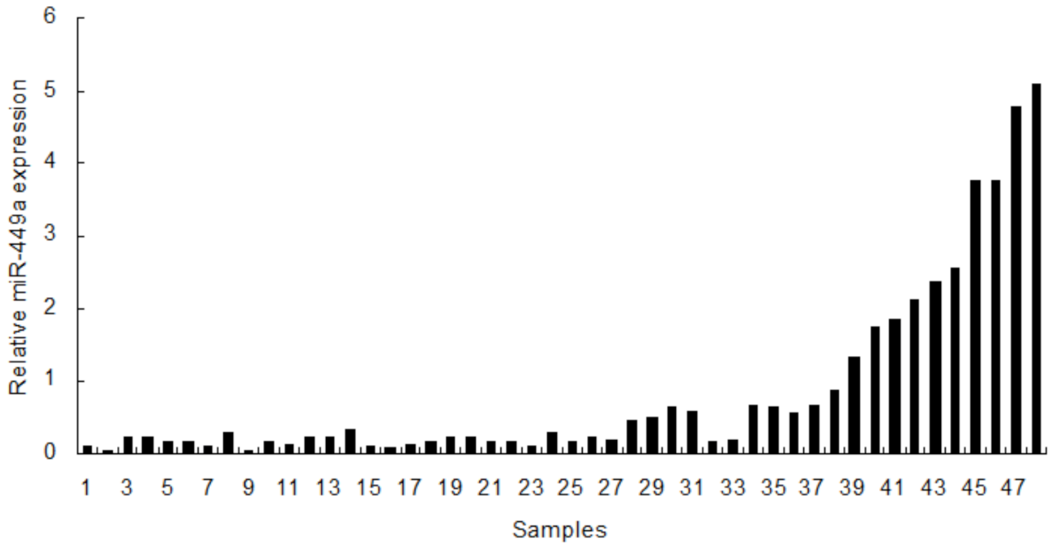

Supplementary Figure S1: miR-449a expression in 48 liver cancer specimens and normal tissues. miRNA abundance was assayed by real time RT-PCR and normalized to U6 RNA.

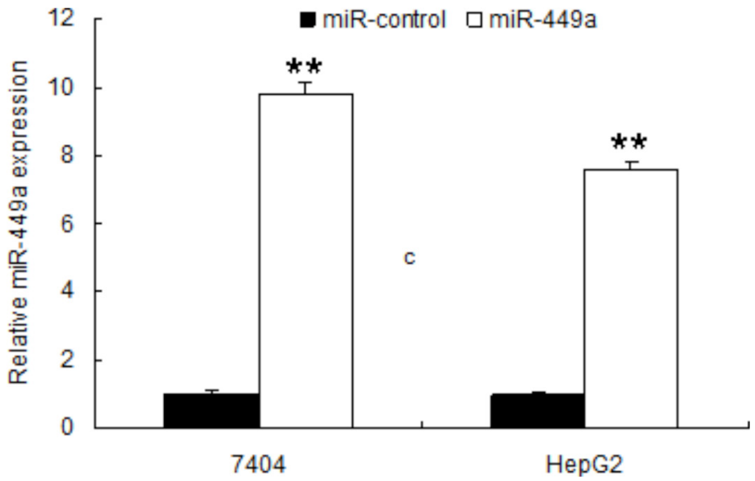

Supplementary Figure S2: A. miR-449a expression in 7404 and HepG2 cells with miR-449 transfection. miR-449a expression in liver cancer cells was examined by real time RT-PCR. **$p < 0.01$

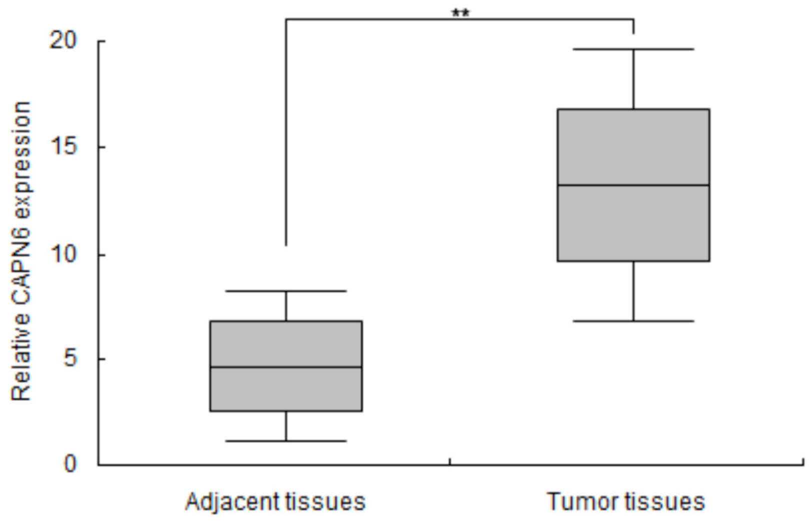

Supplementary Figure S3: CAPN6 mRNA expression in 48 liver cancer specimens and their compared tissues (adjacent tissues). **$p < 0.01$

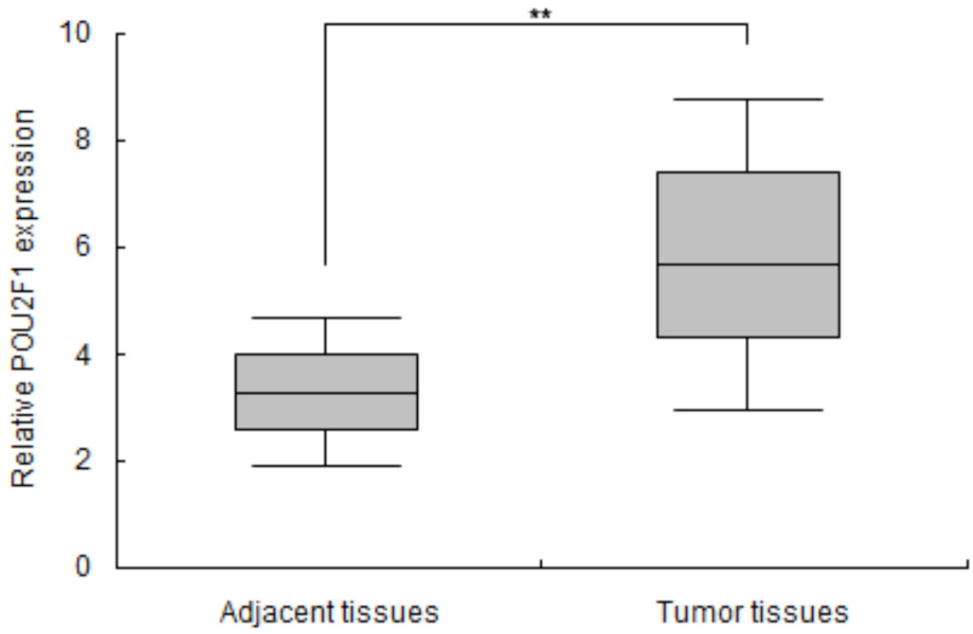

Supplementary Figure S4: POU2F1 mRNA expression in 48 liver cancer specimens and their compared tissues (adjacent tissues). $^{**}p < 0.01$

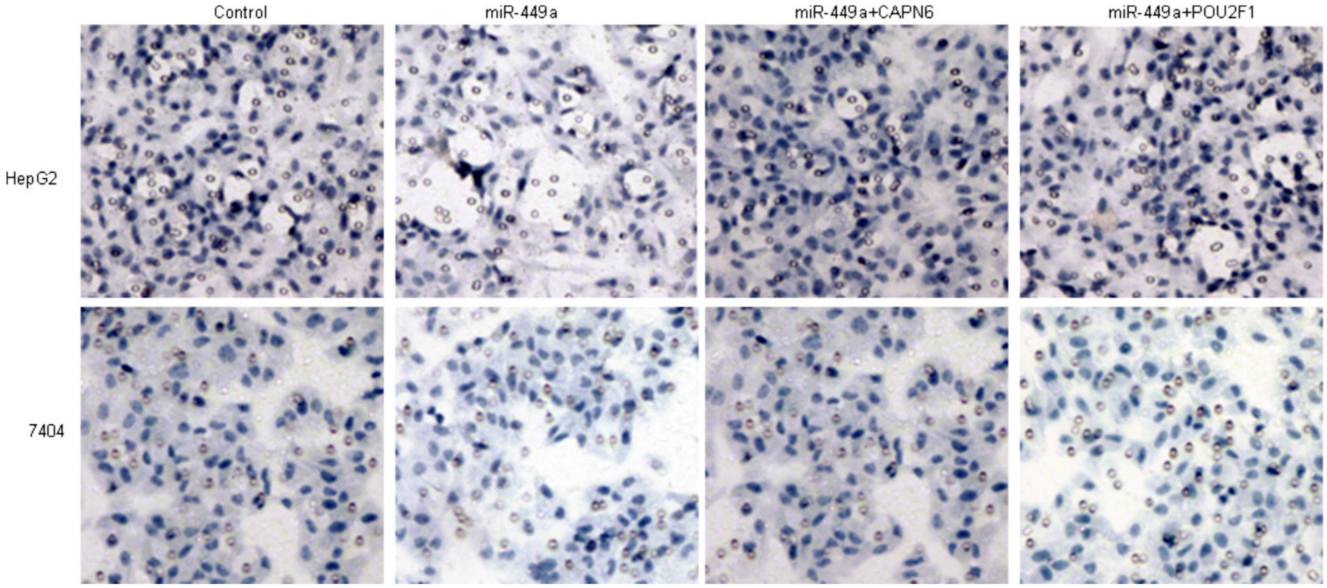

Supplementary Figure S5: miR-449a shows no inhibition of invasion via CAPN6 and POU2F1 in liver cancer cells. Cell invasion was assayed by transwell champer. miR-449a could inhibit HepG2 cell invasion, but not through CAPN6 or POU2F1. There was no inhibition in 7404 cells.
